# Informing health professions education through modern educational theories: a necessary process with potential pitfalls

**DOI:** 10.11604/pamj.2020.36.236.23196

**Published:** 2020-07-30

**Authors:** Mohammed Alhassan

**Affiliations:** 1 Pediatric Department, College of Medicine, Prince Sattam Bin Abdulaziz University, Alkharj, 11942, Saudi Arabia

**Keywords:** Medical education, health professions education, undergraduate, learning theory

## Abstract

This article presents an overview of educational theories focusing on undergraduate health professions education. It represents yet another attempt at untangling the complex issues of what learning theories are about, and how they can inform health professions education. The roles of “self-directed learning” and social interaction in undergraduate medical education are critically discussed; two relevant educational principles are proposed.

## Essay

When a novice embarks on exploring the vast world of educational theories, it is not uncommon for them to feel overwhelmed and find themselves questioning: is educational theory merely a complex, sophisticated, and perplexing description of an endeavor to explain an already evident concept? Indeed, in some instances, the reader may even find themselves forced to question if certain learning theories have actually been intentionally twisted and molded to suit faintly relevant and axiomatic medical-educational concepts and principles that an author was desperate to find a theoretical basis for. At this initial stage of learning about the educational theories, a well-written statement would come to provide great relief. This is the one that captures how we should inform ourselves about the best way to educate/learn: principles and practices in health professions education should “flow partly from psychological theories of learning and partly from pragmatic observation” [1]. Such a statement would save us, the medical educationalists, from falling into the captivity of what can be in some circumstances inherently defective and restraining *mono*-views of *individual* educational theories. As one dives deeper into the published literature on educational theories, it quickly becomes apparent that no one theory or a combination of theories can even come close to fully explaining how we learn or how we should teach.

Nevertheless, “there is nothing as practical as a good theory” [2]. A properly contextualized, appropriately combined, and critically applied educational theory will righteously inform a medical teacher into a better way to teach and to help his students learn [3]. The benefits extend far beyond improving individual practices to inform grand endeavors such as curriculum design and reform. It goes without saying that misapplied or superficially understood theories would be counterproductive. Before instituting any examples of how educational theories can elicit principles, which in turn can guide the educational process, we first need to be precise on what we mean by these terms. “Education theory is the theory of the purpose, application, and interpretation of education and learning. It is largely an umbrella term, being comprised of a number of theories, rather than a single explanation of how we learn, and how we should teach” [4]. One or more educational concepts, models, or principles can be derived from one or more educational theories. “Principle” as a term is thought of as meaning a general truth, a guiding norm [5], or a comprehensive law that governs, in this situation, our endeavors to teach and learn. In education, and as Treacy (1963) depicted them, “principles are generalizations that serve as bases or foundations of educational policies and practices” [6]. While there are only a small number of major (umbrella) educational theories (e.g. cognitivism, constructivism, social learning theory), several educational concepts and models (e.g. self-directed learning, experiential learning, reflective practice) have been derived as a reflection of one or more of these theories to explain and guide different aspects of learning. For example, “workplace-based learning has elements of sociocultural theory; experiential learning has elements of constructivism and humanism theories” [7]. This commentary will try to briefly review a few educational theories, critically discuss the educational concepts derived from them, and elicit two principles that would hopefully guide our educational practices, with particular relevance to undergraduate medical education.

**Self-directed learning or “directed self-learning”:** self-directed learning (SDL) may be defined as a learning model in which the learner can take charge of his own learning process. This involves diagnosing learning needs, identifying learning goals, selecting learning strategies, and evaluating learning performances and outcomes [8]. Again, as noted above, self-directed learning is not an educational theory per se. Instead, it can be viewed as a concept that has its roots originating from several learning theories, as well as from research-supported observations [9]. Humanistic and cognitive approaches may provide the primary theoretical cover for self-directed learning [7], though social learning and constructivism learning theories have their essential contributions as well. The humanistic theory approaches the learner as a human being with a conscious free will and self-motivation to learn/improve in order to reach “self-actualization” [10]. Cognitivism deals with how learners perceive, process, store (memorize), and retrieve information, and how this changes/restructures the internal “schema” of the learner [11]. Constructivism theorizes that learner constructs new knowledge on a framework of previous experiences and knowledge bases [12]. This new knowledge will, in turn, affect how the learner acquires and processes future knowledge. Social theories of learning inspect the effect of social activities on learning, arguing that some aspects of learning cannot be achieved on an individual level without social interaction [1].

Self-directed learning, thus, is neither synonymous with independent learning nor equal to self-paced studying. Rather, it is a broader concept that incorporates, also, the ability to diagnose the learning gaps and to consequently set the learning goals, the skill to discern how to close these gaps, and to determine the validity and reliability of available learning resources. All these should interact within a milieu of attributes such as self-motivation, self-discipline, persistence, curiosity, self-reflection, among others [13]. SDL has gained its popularity in medical education through a body of research that demonstrated its efficacy and effectiveness as a learning model [14,15], in addition to its apparent cost-effectiveness [16]. Compared with traditional teaching methods, SDL was shown to be moderately superior. However, this superiority may be limited to the cognitive domain with no significant advantages in the psychomotor nor in the affective domain [17]. The teacher´s role in SDL moves gradually from authoritative/instructor to a facilitator or even a consulting one [18].

There are critical pitfalls to avoid, however, where SDL is to be employed successfully. While “advanced learners seemed to benefit more from SDL” [17], a carelessly adopted SDL can be enormously counterproductive to the novice learner. A brilliant medical student could readily go astray in the learning path with devastating consequences in an educational environment where SDL is loosely applied without its accompanying guiding principles: i) “Too little direction will result in confusion and inefficient and ineffective learning” [19]. It is evident that SDL is not a rigid concept of learning. Rather it is a multi-staged concept [18], a hierarchy in which the learner should move seamlessly from a hugely dependent to a totally self-directed one. Each stage suits a specific level of learner but may not be suitable for another. While the higher stages of self-direction in Grow´s model may fit a qualified doctor in a “continuous professional development/continuous medical education” manner, applying them to a medical student may make collateral damage. ii) Directing students to suitable learning resource materials is a must. A vivid memory of the author is of two brilliant medical student colleagues. They insisted on using huge reference textbooks to study physiology and biochemistry during the 2^nd^ year of medical school. Both failed the final examination, as could be expected, while the rest of us who studied to-the-point lecture notes, passed smoothly. Medical students, especially in the early years, should be directed to carefully prepared and quality assured resources. A well-designed study guide should also be provided to them. iii) Intended learning outcomes should be explicitly communicated to medical students to guide their SDL. A “directed self-learning” (DSL) approach [1,19] was proposed as a modification to the SDL to provide more guidance and direction to the medical students´ learning process while allowing them to enjoy some form of autonomy and control over their study time and pace as well as their choice of preferred learning style.

**Social constructivism:** “social interaction is important in the knowledge construction process” [20]. This “second-wave” constructivism theory, pioneered by Vygotsky (as cited by Kaufman in Cantillon & Wood, 2010 [21], argues that some “zones” of learning can be difficult, if not impossible, to construct without social encounters with a more competent or knowledgeable group that may include, in medical education respect, peers as well as the teacher. The more capable members in a social context will help “scaffold” the student into a new range of understandings and skills [22]. Consequently, the teacher´s role here is a facilitator who firstly should help students explore and reaffirm their prior knowledge and experiences upon which new knowledge can construct, and secondly, to ensure the social learning event is going in the right direction. This social learning, however, can frequently take place casually (in a non-curricular manner), and this should also be encouraged by educators. Formal learning activities such as problem-based learning, team-based learning, and flipped classroom (but not traditional lecturing) effectuate this theory on the ground. It may also be deduced by now that the much desirable “active learning” where “both the instructor and the students work cooperatively” [23] is implicit within social constructivism.

## Conclusion

The following are two suggested guiding principles for undergraduate medical education as concluded from the above discussion: 1) Medical students should be given necessary and tapering amount of guidance, support, and direction appropriate to their educational level while allowing them to enjoy and build-up some autonomy and control over their study time and pace as well as their choice of preferred learning strategy, style, and resources (directed self-learning). 2) Medical students should be exposed to challenging problems and tasks they may not be able to solve individually, and then should be encouraged to work them out through interaction with teachers and peers.
